# Association of age-related hearing loss, tinnitus, and chronic low back pain in middle-aged and older Korean adults

**DOI:** 10.1371/journal.pone.0291396

**Published:** 2023-09-08

**Authors:** Hye-Mi Noh, Yi Hwa Choi, Jee Hye Wee, Hong Ji Song, Hye-Ji An, Keum Ji Kim, Soo Kyung Lee, Min Soo Jang, Nayoung Yeon

**Affiliations:** 1 Department of Family Medicine, Hallym University Sacred Heart Hospital, College of Medicine, Hallym University, Anyang, Korea; 2 Department of Anesthesiology and Pain Medicine, Hallym University Sacred Heart Hospital, College of Medicine, Hallym University, Anyang, Korea; 3 Department of Otorhinolaryngology-Head and Neck Surgery, Hallym University College of Medicine, Anyang, Korea; Aichi Prefectural Mikawa Aoitori Medical and Rehabilitation Center for Developmental Disabilities, JAPAN

## Abstract

This was a cross-sectional study conducted to evaluate the association between hearing impairment and low back pain (LBP) using data from the Korean National Health and Nutrition Examination Survey. A total of 5,504 middle-aged and older Korean adults (aged ≥50 years old) who underwent plain radiography of the lumbar spine and pure tone audiometry were included. The presence of LBP was evaluated using a questionnaire, which included a question on whether the patient had experienced LBP that lasted for more than 30 days during the past three months. Patients with age-related hearing loss (ARHL) were defined as those with bilateral hearing impairment who met the following criteria: 1) normal otologic examination results, 2) average pure-tone hearing thresholds of ≤15 dB in both ears, and 3) no occupational noise exposure. Multivariable logistic regression analysis showed that ARHL was not associated with LBP (odds ratio, 1.33; 95% CI, 0.94–1.89) after adjusting for potential confounders in the final model. However, when participants without both ARHL and tinnitus were defined as the reference group, the results showed that the participants with both ARHL and tinnitus were more likely to have LBP (OR, 1.86; 95% CI, 1.11–3.11). These results indicate that ARHL with tinnitus is significantly associated with LBP. We recommend that elderly patients with ARHL and tinnitus increase their daily physical activities and engage in more muscle-strengthening exercises to prevent LBP.

## Introduction

The World Health Organization reported that approximately 432 million adults have disabling hearing loss, and that more than 5% of the world population requires rehabilitation for hearing loss [1]. The incidence of age-related hearing loss (ARHL) in Korea is projected to increase about twice as much in the next 20 years [2]. ARHL can cause social isolation attributable to decreased physical function by impairing effective communication and inducing depression and possible cognitive impairment [3, 4].

Low back pain (LBP) is the most common musculoskeletal symptom experienced by older patients [5]. Factors such as age, sex, genetic predisposition, occupation, smoking, race, job satisfaction, psychological issues, anthropometry, and posture are associated with non-specific back pain [6]. Hearing loss is not a primary risk factor for LBP. However, older patients with back pain and hearing loss are frequently encountered in clinical practice, and communication difficulties during consultation may result in refractory chronic pain. Older patients with mild or severe hearing impairment are often unable to maintain an exercise regimen due to cognitive impairment caused by reduced auditory stimulation and disrupted social interactions. In addition, the severity of hearing loss is linearly associated with the risk of dementia and cognitive dysfunction measured using the Mini-Mental State Examination scale [7, 8]. Further, correlations between ARHL and the potential risk factors for chronic LBP, such as sarcopenia or osteoporosis, have been reported [9, 10].

Hearing loss is often accompanied by tinnitus, and the prevalence of tinnitus increases with aging [11]. Previous studies have indicated that tinnitus is associated with chronic pain [12, 13]. Therefore, we propose that ARHL or tinnitus may be associated with intractable LBP among older adults. Thus, the aim of this study was to evaluate the association between ARHL, tinnitus, and LBP in older adults using a nationally representative sample of middle-aged and older Korean adults.

## Methods

### Study population

**Fig 1:**
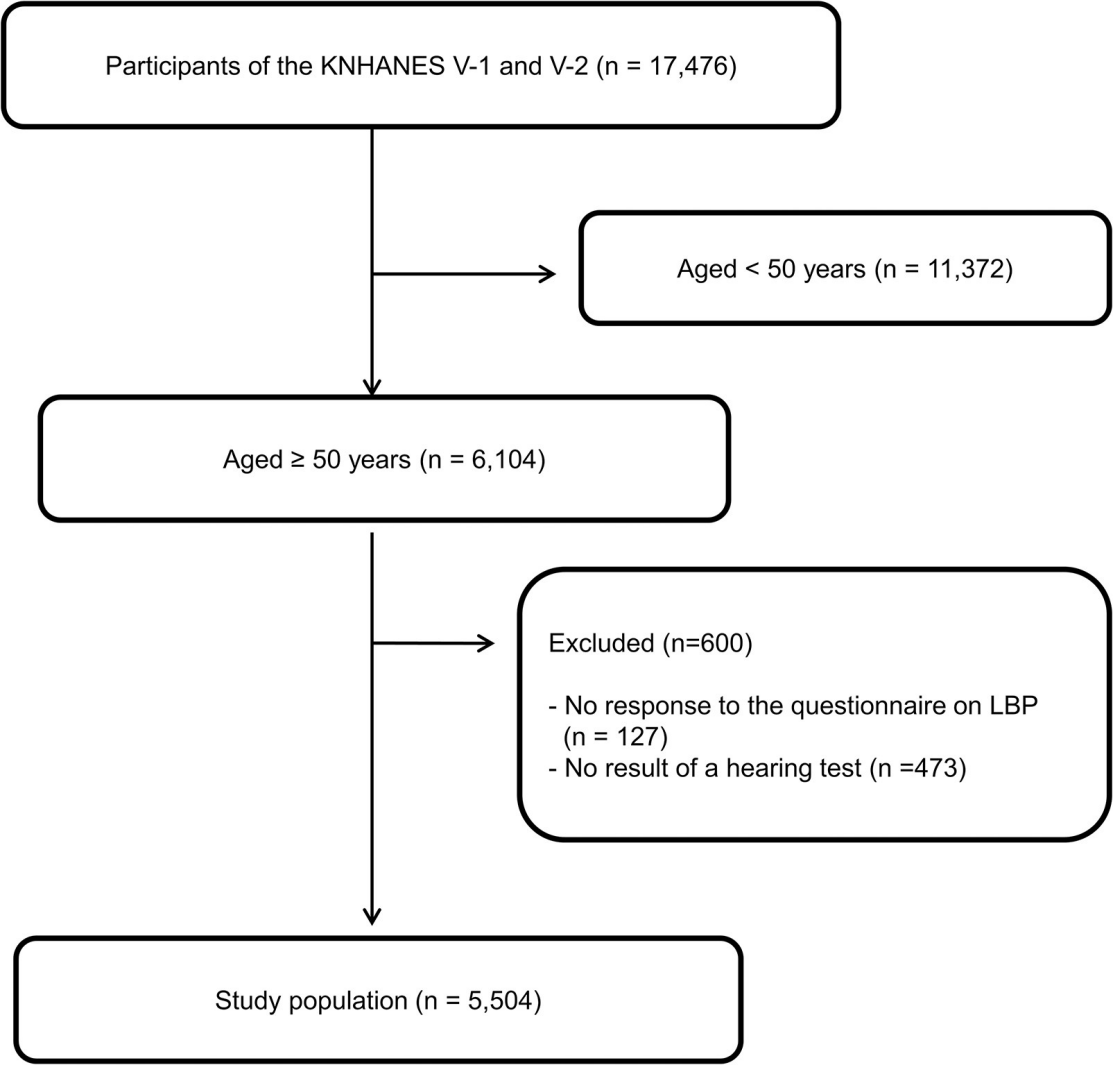
Study flow diagram.

We used data from the 2010–2011 cycle of the Korean National Health and Nutrition Examination Survey (KNHANES V-1 and V2). The KNHANES has been conducted annually by the Korea Disease Control and Prevention Agency (KDCA) since 1998. We selected 6,104 participants (aged ≥50 years old) who underwent plain radiography of the lumbar spine for this study. The presence of LBP was evaluated using a questionnaire. We excluded participants who did not respond to the question on LBP in the questionnaire (n = 127) and those that did not undergo a hearing test (n = 473). Finally, 5,504 individuals were included in the study (Fig 1).

All procedures were approved by the ethics committee of the KDCA (Institutional Review Board numbers: 2010-02CON-21-C and 2011-02CON-06-C). Informed consent was obtained from all the KNHANES participants. Requirement for the informed consent in the present study was waived by the board owing to the retrospective nature of the study. The KNHANES data are publicly available on the KNHANES website (https://knhanes.cdc.go.kr/knhanes/eng/index.do).

### LBP

LBP was defined as responding “yes” to the question, “Have you had LBP for more than 30 days in the past three months?”

### Audiometric measurements

Otorhinolaryngologists performed hearing tests in a soundproof booth using a diagnostic audiometer (SA-203; Entomed, Malmoe, Sweden). The test was performed at frequencies of 0.5, 1, 2, 3, 4, and 6 kHz. The otorhinolaryngologists also performed otologic examinations using a 4-mm 0° endoscope (Xion GmbH, Berlin, Germany) to evaluate the tympanic membrane or middle ear. Hearing impairment was defined as a pure tone threshold greater than an average of 25 dB at 0.5, 1, 2, and 4 kHz [14]. We categorized the patients into those with normal hearing, unilateral hearing impairment, and bilateral hearing impairment. Patients with ARHL were defined as patients with bilateral hearing impairments who met the following criteria: 1) normal otologic examination results, 2) average pure-tone thresholds of ≤15 dB in both ears, and 3) no occupational noise exposure. The severity of hearing impairment was defined based on the hearing threshold of the better ear as follows: mild, 26–40 dB; moderate, 41–70 dB; and severe, >71 dB [15].

### Definitions of subjective hearing impairment and tinnitus

We defined subjective hearing impairment as having test results that indicate normal hearing but having difficulty hearing. Participants were asked to the following question to estimate their hearing ability: “Please select the statement that best describes your hearing (without hearing aids): 1) no difficulty, 2) a little difficulty, 3) a lot of difficulty, and 4) I can’t hear at all.” Patients who selected 2), 3), or 4) were defined as those with hearing difficulties. Tinnitus was assessed using the following question: “In the past year, have you experienced any noise in your ears (crackling, beeping, humming, machinery, etc.)?”. Participants who answered “yes” to the question were defined as patients with tinnitus.

### Other variables

The following demographic data were collected: age, sex, education, household income, occupation, occupational noise exposure, monthly alcohol consumption (< once vs. ≥ once/month), smoking status (ex-smoker and current smoker), and physical activity (muscle-strengthening exercises or walking physical activity). Engagement in muscle-strengthening exercises was assessed using the following question: “In the past week, how many days did you engage in strength training exercises, such as push-ups, sit-ups, or lifting dumbbells, weights, or barbells?” Engagement in walking physical activity was assessed by asking participants the following question: “How many days in the past week did you walk for at least 10 minutes at a time, and on those days, how long do you usually walk during the day?” Regular muscle-strengthening exercise was defined as performing strength exercises at least twice a week. Regular walking physical activity was defined as walking for at least 30 minutes for five days a week. Comorbidities (hypertension, diabetes mellitus, chronic kidney disease, rheumatoid arthritis, and depression) were identified using self-report questionnaires. Plain radiographs of the lumbar spine were obtained using an SD 3000 Synchro Stand (Accele Ray SYFM Co., Seoul, Korea). Musculoskeletal radiologists evaluated the severity of osteoarthritis in facet joints according to the Kellgren–Lawrence grading system. The radiologic grade was classified using the method proposed by Yoshimura et al. [16]: 0, normal (no abnormalities including slight osteophytes); 1, suspicious (clear osteophytes); and 2, abnormal (stenosis, osteosclerosis, and large osteophytes). Bone mineral density and body composition were measured using DEXA (DISCOVERY-W, Hologic Inc., Marlborough, MA, USA). Appendicular skeletal muscle mass (ASM) was defined as the muscle mass of the bilateral upper and lower extremities, and the ASM index was calculated as ASM/(height [m])^2^ [17]. Limitation of activity was defined as answering “yes” to the question “In the past month, have you had a day when you had to spend almost the entire day in bed due to illness or injury?”. Dietary intake was assessed using a 24 h dietary recall method, and total energy and nutrient intake were calculated according to the National Standard Food Composition Table [18].

### Statistical analysis

We performed complex-sample analysis because the KNHANES data were extracted using a two-stage stratified cluster sampling method. Continuous variables are presented as mean ± standard error, whereas categorical data are presented as the estimated percentage (standard error). The t-test and χ^2^ test were used to compare the general characteristics of the patients and the prevalence of hearing impairments according to the presence or absence of LBP. Multivariable logistic regression analysis was performed to examine the association between hearing impairment and LBP. Age and sex were adjusted in Model 1. Socioeconomic status (household income, occupation, and education) and lifestyle factors (smoking status, alcohol consumption, and muscle-strengthening exercise) were additionally included in Model 2. ASM index, osteoporosis, Kellgren–Lawrence grading of the lumbar spine, and comorbidities were also included in Model 3. Tinnitus, use of a hearing aid or cochlear implant, limitation of activity, and dietary intake of protein, retinol, riboflavin, and niacin were added to the final model (Model 4). All statistical analyses were performed using SPSS software (version 25.0; IBM Corp., Armonk, NY, USA). P < 0.05 was considered statistically significant.

## Results

### General characteristics

Of the 5,504 participants included, 1,414 (25.7%) had LBP. Table 1 shows the general characteristics of the participants categorized according to the presence of LBP. The participants with LBP were older and had a higher proportion of women than the participants without LBP (both P<0.001). Smoking, alcohol consumption, and performing muscle-strengthening exercise were more common among participants without LBP than among those with LBP (P<0.001, 0.001, and <0.001, respectively). The participants with LBP had lower educational levels and household incomes than those without LBP (both P<0.001). Regarding occupation, the proportions of participants with LBP who were unemployed or employed in the agriculture, forestry, and fishery industries were higher than those of the participants without LBP in these occupation categories (P<0.001). Occupational noise exposure was more common in participants without LBP than in those with LBP (P = 0.015). Participants with LBP had a lower ASM index and a higher prevalence of osteoporosis and grade 2 lumbar spine osteoarthritis than those without LBP (P<0.001, 0.001, and 0.015, respectively). Participants with LBP had a higher number of comorbidities, including hypertension, chronic kidney disease, rheumatoid arthritis, and depression, than those without LBP (P<0.001, P = 0.03, P<0.001, and P<0.001, respectively).

**Table 1:** Characteristics of the study population.

Variables	Low back pain		P value
	Yes (n = 1,414)	No (n = 4,090)	
Age			<0.001
50–59 years	34.0% (1.8%)	53.7% (1.1%)	
60–69 years	30.2% (1.5%)	27.6% (0.8%)	
70–79 years	27.4% (1.5%)	16.1% (0.7%)	
≥ 80 years	8.4% (1.0%)	2.6% (0.3%)	
Sex			<0.001
Male	26.9% (1.5%)	53.1% (0.8%)	
Female	73.1% (1.5%)	46.9% (0.8%)	
BMI (kg/m)	24.0 (0.10)	24.0 (0.06)	0.992
Smoking			<0.001
Ex-smoker	16.1% (1.2%)	27.8% (0.8%)	
Current smoker	14.9% (1.2%)	21.3% (0.9%)	
Alcohol consumption	47.8% (3.5%)	61.2% (1.7%)	0.001
Muscle-strengthening exercise (≥2 times/week)	15.2% (1.2%)	27.7% (1.0%)	<0.001
Walking physical activity	35.5% (1.8%)	39.4% (1.0%)	0.051
Educational level			<0.001
≤ Elementary school	67.1% (1.7%)	38.6% (1.2%)	
Middle school	14.7% (1.2%)	20.7% (0.8%)	
High school	13.1% (1.1%)	27.6% (0.9%)	
≥College	5.1% (0.8%)	13.1% (1.0%)	
Household income			<0.001
Low	32.3% (1.7%)	24.0% (1.0%)	
Lower middle	27.6% (1.5%)	25.2% (1.0%)	
Upper middle	23.0% (1.5%)	26.2% (0.9%)	
High	17.1% (1.3%)	24.5% (1.1%)	
Occupation			<0.001
Office work	3.3% (0.6%)	10.6% (0.7%)	
Sales and services	8.9% (1.0%)	12.8% (0.8%)	
Agriculture, forestry, and fishery	17.9% (2.2%)	13.5% (1.5%)	
Machine fitting and simple labor	18.9% (1.6%)	23.6% (1.0%)	
Unemployed	50.9% (1.9%)	39.4% (1.2%)	
Occupational noise exposure (%)	10.9% (1.1%)	14.1% (0.9%)	0.015
DEXA			
Appendicular skeletal muscle mass/height^2^	6.31 (0.05)	6.74 (0.03)	<0.001
Osteoporosis	33.5% (1.8%)	16% (0.8%)	0.001
Lumbar spine osteoarthritis			0.015
Normal	5.2% (2.4%)	15.6% (2.7%)	
Grade 1	45.4% (4.6%)	48.0% (3.5%)	
Grade 2	49.4% (4.6%)	36.3% (3.5%)	
Comorbidity			
Hypertension	44.9% (1.7%)	35.6% (1.0%)	<0.001
Diabetes mellitus	16.1% (1.3%)	14.4% (0.7%)	0.215
Chronic kidney disease	0.9% (0.3%)	0.3% (0.1%)	0.03
Rheumatoid arthritis	6.7% (0.8%)	2.8% (0.3%)	<0.001
Depression	7.9% (0.9%)	4.1% (0.4%)	<0.001
Limitation of activity (bed rest in the past month)	19.2% (1.4%)	5.1% (0.5%)	<0.001
Dietary intake			
Total energy intake (kcal/day)	1704.9 (30.25)	1960.9 (18.28)	<0.001
Protein intake (g/day)	55.9 (1.35)	67.2 (0.77)	<0.001
Retinol (μg/day)	57.7 (5.77)	73.9 (2.69)	0.045
Riboflavin (mg/day)	0.93 (0.03)	1.14 (0.02)	0.022
Niacin (mg/day)	13.6 (0.37)	16.2 (0.19)	<0.001

Data are presented as mean ± standard error or estimated percentage (standard error).

### Hearing impairment according to LBP

Table 2 shows the prevalence of hearing impairment in the study population according to the presence of LBP. Unilateral and bilateral hearing impairments were more common in participants with LBP than in those without LBP (P<0.001). ARHL and mild, moderate, and severe hearing impairments were more common in participants with LBP than in those without LBP (both P<0.001). Participants with LBP showed higher mean hearing thresholds at the four frequencies (0.5, 1, 2, and 4 kHz) and high frequencies (4 and 6 kHz) in both ears than those without LBP (both P<0.001). Subjective hearing impairment was more common in participants with LBP than in those without LBP (P = 0.007).

**Table 2:** Hearing impairments and hearing thresholds in the study population categorized according to the presence of low back pain.

Variables	Low back pain		P value
	Yes (n = 1,414)	No (n = 4,090)	
Hearing impairment			<0.001
Normal	45.8% (1.7%)	58.7% (1.0%)	
Unilateral hearing impairment	18.2% (1.2%)	17.2% (0.8%)	
Bilateral hearing impairment	36.0% (1.6%)	24.1% (0.8%)	
Severity of hearing impairment			<0.001
Mild hearing impairment (26–40 dB)	23.8% (1.4%)	17.0% (0.7%)	
Moderate hearing impairment (41–70 dB)	10.5% (1.0%)	6.4% (0.5%)	
Severe hearing impairment (>71 dB)	1.7% (0.4%)	0.7% (0.2%)	
Age-related hearing loss	32.8% (1.9%)	18.3% (0.9%)	<0.001
Hearing thresholds at four frequencies (0.5, 1, 2, and 4 kHz)			
Lt	29.0 (0.56)	24.0 (0.33)	<0.001
Rt	27.8 (0.62)	23.3 (0.33)	<0.001
Hearing thresholds at high frequencies (4 and 6 kHz)			
Lt	48.1 (0.72)	43.2 (0.46)	<0.001
Rt	45.3 (0.78)	41.2 (0.44)	<0.001
Subjective hearing impairment	12.6% (1.7%)	8.4% (0.7%)	0.007
Tinnitus	32.3% (1.5%)	23.9% (0.9%)	<0.001
Use of hearing aid or cochlear implant	1.4% (0.4%)	0.8% (0.2%)	0.083

Data are presented as mean ± standard error or estimated percentage (standard error).

### Multivariable logistic regression analysis of the association between hearing impairment and LBP

Table 3 shows the results of multivariable logistic regression analysis of the association between hearing impairment and LBP. After adjusting for age and sex in Model 1, the adjusted odds of hearing impairment, bilateral hearing impairment, ARHL, and subjective hearing impairment in participants with LBP were higher than those in participants without LBP (odds ratio [OR], 1.3, 1.36, 1.54, and 1.61; 95% CI, 1.09–1.54, 1.11–1.66, 1.21–1.97, and 1.15–2.26; respectively). However, after adjusting for potential confounders in Model 3, only ARHL was significantly associated with LBP (OR, 1.53; 95% CI, 1.11–2.13). ARHL was not associated with LBP in the final model (Model 4) (OR, 1.33; 95% CI, 0.94–1.89).

**Table 3:** Logistic regression analysis of the association between hearing impairment and low back pain.

	Model 1		Model 2		Model 3		Model 4	
	OR	95%CI	OR	95%CI	OR	95%CI	OR	95%CI
Hearing impairment								
Yes	1.3	(1.09–1.54)	1.16	(0.97–1.40)	1.2	(0.95–1.52)	1.17	(0.91–1.50)
Normal	1		1		1		1	
Unilateral hearing impairment								
Yes	1.23	(0.99–1.52)	1.13	(0.91–1.40)	1.14	(0.87–1.50)	1.21	(0.90–1.62)
Normal	1		1		1		1	
Bilateral hearing impairment								
Yes	1.36	(1.11–1.66)	1.19	(0.96–1.48)	1.25	(0.96–1.64)	1.13	(0.85–1.51)
Normal	1		1		1		1	
Age-related hearing loss								
Yes	1.54	(1.21–1.97)	1.39	(1.07–1.81)	1.53	(1.11–2.13)	1.33	(0.94–1.89)
Normal	1		1		1		1	
Subjective hearing impairment								
Yes	1.61	(1.15–2.26)	1.7	(1.19–2.42)	1.52	(0.94–2.46)	1.12	(0.84–1.49)
No	1		1		1		1	

OR, odds ratio; CI, Confidence interval

Model 1: adjusted for age and sex.

Model 2: adjusted for age, sex, household income, occupation, education, smoking status, alcohol consumption, and muscle-strengthening exercise.

Model 3: adjusted for age, sex, household income, occupation, education, smoking status, alcohol consumption, and muscle-strengthening exercise, appendicular skeletal muscle mass index, osteoporosis, Kellgren–Lawrence grading of the lumbar spine, and comorbidities.

Model 4: adjusted for age, sex, household income, occupation, education, smoking status, alcohol consumption, muscle-strengthening exercise, appendicular skeletal muscle mass index, osteoporosis, Kellgren–Lawrence grading of the lumbar spine, comorbidities, tinnitus, use of hearing aid use or cochlear implant, limitation of activity, and dietary intake of protein, retinol, riboflavin, and niacin.

### Multivariable logistic regression analysis of the associations between age-related hearing loss, tinnitus, and LBP

The results of multivariable logistic regression analysis of the associations between age-related hearing loss, tinnitus, and low back pain are presented in Table 4. The results showed that when participants without both ARHL and tinnitus were defined as the reference group, participants with both ARHL and tinnitus were more likely to have LBP (OR, 1.86; 95% CI 1.11–3.11). However, there was no significant difference in the odds of developing LBP between participants with ARHL and no tinnitus and those with tinnitus and no ARHL (OR, 1.37 and 1.47; 95% CI, 0.90–2.07 and 0.97–2.24).

**Table 4:** Associations between age-related hearing loss, tinnitus, and low back pain.

	Model 1		Model 2		Model 3		Model 4	
	OR	95%CI	OR	95%CI	OR	95%CI	OR	95%CI
Age-related hearing loss (+) and tinnitus (+)	1.97	(1.41–2.76)	1.75	(1.22–2.52)	2.06	(1.28–3.32)	1.86	(1.11–3.11)
Age-related hearing loss (+) and tinnitus (-)	1.48	(1.10–2.01)	1.33	(0.98–1.82)	1.45	(0.97–2.16)	1.37	(0.90–2.07)
Age-related hearing loss (-) and tinnitus (+)	1.38	(1.03–1.85)	1.35	(0.99–1.84)	1.47	(0.99–2.17)	1.47	(0.97–2.24)
Age-related hearing loss (-) and tinnitus (-)	1		1		1		1	

OR, odds ratio; CI, Confidence interval

Model 1: adjusted for age and sex.

Model 2: adjusted for age, sex, household income, occupation, education, smoking status, alcohol consumption, and muscle-strengthening exercise.

Model 3: adjusted for age, sex, household income, occupation, education, smoking status, alcohol consumption, and muscle-strengthening exercise, appendicular skeletal muscle mass index, osteoporosis, Kellgren–Lawrence grading of the lumbar spine, and comorbidities.

Model 4 adjusted age, sex, household income, occupation, education, smoking status, alcohol consumption, and muscle-strengthening exercise, appendicular skeletal muscle mass index, osteoporosis, Kellgren–Lawrence grading of the lumbar spine, comorbidities, hearing aid use or cochlear implant, activity limitation, dietary intake of protein, retinol, riboflavin, and niacin.

## Discussion

In this cross-sectional study, unilateral and bilateral hearing impairments were more common in participants with LBP than in those without LBP. In addition, mild, moderate, severe, and ARHL were more common in participants with LBP than in those without LBP. After adjusting for potential confounding factors, ARHL with tinnitus was significantly correlated with LBP.

ARHL is characterized by symmetrical sensorineural loss of hearing at high frequencies, which can be identified in noise [19], resulting in confusion, conversational breakdown, and social isolation, and affecting an individual’s psychosocial status [20]. Specifically, difficulty with speech perception in a noisy environment gradually progresses into difficulty with speech perception in a quiet environment, resulting in communication errors or worse. Poor perception of speech and warning sounds can result in social isolation and decrease in daily physical activities and exercise that could prevent LBP.

Aerobic exercise plays a crucial role in relieving LBP and preventing further damage by increasing blood flow and supply of nutrients to the soft tissues in lumbar spine structures, which improves the healing process in damaged tissues and reduces stiffness [21]. Muscle strengthening exercises increase strength and control of the trunk muscles to improve spinal instability attributable to decreased physical disability and trunk muscle inactivity in patients with LBP [22]. The findings of this study are in line with those of previous studies that showed that people without LBP engage in muscle-strengthening exercises more often than those with LBP (P<0.001). Physical activity increases aerobic capacity and muscle strength around the spine. However, older people with ARHL may not engage in physical activity regularly owing to difficulties with daily communication and social isolation [23, 24]. Reduced physical activity is associated with an increased incidence of chronic LBP. Moreover, walking at a slow speed is insufficient for rebuilding atrophied muscles in older people, making the development of LBP inevitable [25].

Loss of proprioception resulting in alteration in lumbar spine motion can contribute to LBP [26]. Proprioception allows for perception of the static and dynamic position of the body, and is crucial for controlling movement. Auditory input is as important as visual, vestibular, and proprioceptive inputs for self-perception of movements [27]. Hearing loss does not allow for dynamic tracking of body position for engagement in daily physical activities. Bang et al. [28] reported that moderate hearing loss is associated with postural instability in persons aged 40 years or older. In addition, Shayman et al. [29] suggested that hearing aids and cochlear implants improve gait performance through the improvement of static balance. In the present study, ARHL and tinnitus were more common in patients with LBP than in those without LBP. Our findings could be explained by the abovementioned associations reported in previous studies. The results of the present study suggest that reduced engagement in muscle-strengthening exercises and physical activities secondary to loss of proprioception caused by ARHL may lead to LBP.

Tinnitus is common in older adults, and it can occur as a result of hearing loss [30]. A previous study indicated that tinnitus is associated with chronic pain, and that both tinnitus and pain are subjective symptoms. The study also indicated that noise sensitivity is correlated with generalized sensitivity [13]. ARHL with tinnitus can make speech comprehension and conversation more difficult than ARHL without tinnitus [31].

In this study, we also evaluated the association between subjective hearing impairment and LBP. Subjective hearing impairment was more common in participants with LBP than in those without LBP (P = 0.007). In clinical practice, some patients with intractable LBP who present with subjective hearing impairment may not communicate effectively during treatment due to a lack of concentration or misunderstanding during conversation. A previous study of the Korean population identified that subjective hearing impairment is the main factor associated with decreased quality of communication in the elderly [32]. The first sign of sensorineural hearing loss is difficulty hearing in noisy environments. Compared with high frequency hearing impairment, difficulty with comprehension during conversation leads to low discrimination scores in the speech-in-noise test [33].

The results of this study showed that lower ASM index, osteoporosis, and lumbar spine osteoarthritis were associated with LBP, a finding that is in line with the results of previous studies. Age-related loss of muscle mass and fat degeneration contribute to chronic LBP in older patients owing to the sarcopenic changes in trunk muscles that occur with increasing age [34]. In addition, osteoporosis causes reduction in bone mass and micro-architectural deterioration of bony structures, which causes bones to become fragile and increases the risk of fractures, leading to reduction in spinal height and development of a hunched posture that makes the individual susceptible to LBP [35]. Spinal degeneration is also associated with LBP and reduced physical function. Goode et al. [36] reported that spinal osteoarthritis is associated with pain, disability, and reduced function, and should not be ignored. The progressive loss of muscle mass, bone loss, and spinal degeneration in older adults caused by sarcopenia, osteoporosis, and lumbar osteoarthritis lead to postural changes and reduced physical activity, which in turn lead to the development of chronic LBP.

Some older people consider hearing loss as a part of the aging process and do not consider seeking treatment. When untreated or under-treated patients with ARHL visit a clinic for the treatment of LBP, they often cannot communicate effectively for clinicians to explain disease progression, course of treatment, and preventive measures; therefore, they show a tendency towards intractable neuropathic pain. Female healthcare providers who speak at a higher pitch are more likely to have difficulties communicating with older people in clinical practice because ARHL is characterized by progressive sensorineural hearing loss, especially at high frequencies. Appropriate correction and prevention of hearing impairment should be encouraged to reduce the risk of intractable LBP in older adults.

This study had several limitations. First, given that we used cross-sectional data (KNHANES) for this study, the causal relationship between hearing loss with tinnitus and LBP was unclear. Second, we evaluated the frequency of performing muscle-strengthening exercises using the question “In the past week, how many days did you perform strength training exercises such as push-ups, sit-ups, or lifting dumbbells, weights, or barbells?”. The questionnaire did not include additional questions on the intensity or duration of the exercises. If differences in effect of ARHL with tinnitus on LBP according to the intensity or duration of exercises were known, a more specific explanation of the mechanism underlying the association between ARHL with tinnitus and LBP would have been provided. Third, age is a top-coding category in the KNHANES, and the ages of all participants older than 80 years are coded as 80 years. In the statistical analysis, we divided age into four categories at intervals of 10 years. Therefore, whether our results can be applied to adults older than 80 years is unclear. A further prospective study that includes participants in a wider range of age groups is necessary to clarify the association between ARHL with tinnitus and LBP in the middle-aged and older population.

## Conclusion

This study demonstrated that ARHL with tinnitus is significantly associated with LBP. The results of this study indicate that untreated hearing loss with tinnitus in middle-aged and older adults can increase the risk of intractable LBP. ARHL and tinnitus may be crucial factors associated with intractable LBP in elderly persons. Elderly patients with ARHL and tinnitus should be encouraged to increase their daily physical activities and engage in more muscle-strengthening exercises to prevent LBP.

## Supporting information

S1 Checklist STROBE statement—checklist of items that should be included in reports of observational studies. (DOCX)
