# Supplementary material for: Association of age-related hearing loss, tinnitus, and chronic low back pain in middle-aged and older Korean adults
Source: PLoS One. 2023 Sep 8;18(9):e0291396. doi: 10.1371/journal.pone.0291396 (PMC10490886; doi:10.1371/journal.pone.0291396)
Supplement: S1 Checklist — (DOCX) [file pone.0291396.s001.docx]

STROBE Statement—checklist of items that should be included in reports of observational studies

|  | Item No. | Recommendation | Page  No. | Relevant text from manuscript |
| --- | --- | --- | --- | --- |
| **Title and abstract** | 1 | (*a*) Indicate the study’s design with a commonly used term in the title or the abstract | 2 | This cross sectional study… |
|  |  | (*b*) Provide in the abstract an informative and balanced summary of what was done and what was found | 2 | In conclusion, age-realated hearing loss was… |
| Introduction | | | |  |
| Background/rationale | 2 | Explain the scientific background and rationale for the investigation being reported | 3 | Older patients with hearing … |
| Objectives | 3 | State specific objectives, including any prespecified hypotheses | 3 | We propose that ARHL may be. |
| Methods | | | |  |
| Study design | 4 | Present key elements of study design early in the paper | 4 | LBP, Audiometric measurement |
| Setting | 5 | Describe the setting, locations, and relevant dates, including periods of recruitment, exposure, follow-up, and data collection | 4 | The KNHANES has been conducted by…. |
| Participants | 6 | (*a*) *Cohort study*—Give the eligibility criteria, and the sources and methods of selection of participants. Describe methods of follow-up  *Case-control study*—Give the eligibility criteria, and the sources and methods of case ascertainment and control selection. Give the rationale for the choice of cases and controls  *Cross-sectional study*—Give the eligibility criteria, and the sources and methods of selection of participants | 4 | We selected 6,104 participants who underwent plain radiography of the lumbar spine. |
|  |  | (*b*) *Cohort study*—For matched studies, give matching criteria and number of exposed and unexposed  *Case-control study*—For matched studies, give matching criteria and the number of controls per case | (-) | (cross-sectional study) |
| Variables | 7 | Clearly define all outcomes, exposures, predictors, potential confounders, and effect modifiers. Give diagnostic criteria, if applicable | 5 | Other variables |
| Data sources/ measurement | 8* | For each variable of interest, give sources of data and details of methods of assessment (measurement). Describe comparability of assessment methods if there is more than one group | 4,5,6 | Questionnaire on LBP and hearing test. |
| Bias | 9 | Describe any efforts to address potential sources of bias | 14 | The causal relationship was… |
| Study size | 10 | Explain how the study size was arrived at | 4 | Finally, 5,504 individuals were.. |

Continued on next page

| Quantitative variables | 11 | Explain how quantitative variables were handled in the analyses. If applicable, describe which groupings were chosen and why | 6,7 | We used complex sample analysis.. |
| --- | --- | --- | --- | --- |
| Statistical methods | 12 | (*a*) Describe all statistical methods, including those used to control for confounding | 6 | T-test and χ^2^ test were conducted... |
|  |  | (*b*) Describe any methods used to examine subgroups and interactions | 6,7 | Multivariate logistic regression… |
|  |  | (*c*) Explain how missing data were addressed | 4 | We excluded the participants.. |
|  |  | (*d*) *Cohort study*—If applicable, explain how loss to follow-up was addressed  *Case-control study*—If applicable, explain how matching of cases and controls was addressed  *Cross-sectional study*—If applicable, describe analytical methods taking account of sampling strategy | 4 | We selected 6,104 participants who underwent plain radiography of the lumbar spine. |
|  |  | (*e*) Describe any sensitivity analyses | 6 | Multivariable logistic regression … |
| Results | | | | |
| Participants | 13* | (a) Report numbers of individuals at each stage of study—eg numbers potentially eligible, examined for eligibility, confirmed eligible, included in the study, completing follow-up, and analysed | 4 | We selected 6,104 participants… |
|  |  | (b) Give reasons for non-participation at each stage | 4 | We excluded the participants .. |
|  |  | (c) Consider use of a flow diagram | Fig 1. | Attached |
| Descriptive data | 14* | (a) Give characteristics of study participants (eg demographic, clinical, social) and information on exposures and potential confounders | 7,8  (Table 1) | Table 1 shows the general characteristics of the participants… |
|  |  | (b) Indicate number of participants with missing data for each variable of interest | 4. | We excluded the participants…. |
|  |  | (c) *Cohort study*—Summarise follow-up time (eg, average and total amount) |  |  |
| Outcome data | 15* | *Cohort study*—Report numbers of outcome events or summary measures over time |  |  |
|  |  | *Case-control study—*Report numbers in each exposure category, or summary measures of exposure |  |  |
|  |  | *Cross-sectional study—*Report numbers of outcome events or summary measures | 9,10 | Participants with LBP had a higher. |
| Main results | 16 | (*a*) Give unadjusted estimates and, if applicable, confounder-adjusted estimates and their precision (eg, 95% confidence interval). Make clear which confounders were adjusted for and why they were included | 9 | Participants with LBP and a higher proportion of unilateral and bilateral hearing impairment … |
|  |  | (*b*) Report category boundaries when continuous variables were categorized | 10 | After adjusting for age and sex… |
|  |  | (*c*) If relevant, consider translating estimates of relative risk into absolute risk for a meaningful time period | 11  (Table 2) | Logistic regression analysis of hearing impairment and LBP. |

Continued on next page

| Other analyses | 17 | Report other analyses done—eg analyses of subgroups and interactions, and sensitivity analyses | 10 | After accounting for potential …. |
| --- | --- | --- | --- | --- |
| Discussion | | | | |
| Key results | 18 | Summarise key results with reference to study objectives | 12 | In this cross-sectional study, …. |
| Limitations | 19 | Discuss limitations of the study, taking into account sources of potential bias or imprecision. Discuss both direction and magnitude of any potential bias | 14, 15 | This study had several limitation. |
| Interpretation | 20 | Give a cautious overall interpretation of results considering objectives, limitations, multiplicity of analyses, results from similar studies, and other relevant evidence | 12,13 | Our findings are in line with previous studies which found…. |
| Generalisability | 21 | Discuss the generalisability (external validity) of the study results | 14, 15 | The causal relationship between… |
| Other information | |  | | |
| Funding | 22 | Give the source of funding and the role of the funders for the present study and, if applicable, for the original study on which the present article is based | 16 | This research received no external funding. |

*Give information separately for cases and controls in case-control studies and, if applicable, for exposed and unexposed groups in cohort and cross-sectional studies.

**Note:** An Explanation and Elaboration article discusses each checklist item and gives methodological background and published examples of transparent reporting. The STROBE checklist is best used in conjunction with this article (freely available on the Web sites of PLoS Medicine at http://www.plosmedicine.org/, Annals of Internal Medicine at http://www.annals.org/, and Epidemiology at http://www.epidem.com/). Information on the STROBE Initiative is available at www.strobe-statement.org.
